# The Synthesis and Initial Evaluation of MerTK Targeted PET Agents

**DOI:** 10.3390/molecules27051460

**Published:** 2022-02-22

**Authors:** Li Wang, Yubai Zhou, Xuedan Wu, Xinrui Ma, Bing Li, Ransheng Ding, Michael A. Stashko, Zhanhong Wu, Xiaodong Wang, Zibo Li

**Affiliations:** 1 Biomedical Research Imaging Center, Department of Radiology, and UNC Lineberger Comprehensive Cancer Center, The University of North Carolina at Chapel Hill, Chapel Hill, NC 27514, USA; liwang_512@163.com (L.W.); xuedanwu@ad.unc.edu (X.W.); xinrui_ma@med.unc.edu (X.M.); zibo_li@med.unc.edu (Z.L.); 2 Center for Integrative Chemical Biology and Drug Discovery, Division of Chemical Biology and Medicinal Chemistry, Eshelman School of Pharmacy, The University of North Carolina at Chapel Hill, Chapel Hill, NC 27599, USA; zhouyb@email.unc.edu (Y.Z.); libingchem@gmail.com (B.L.); rd840@georgetown.edu (R.D.); mstashko@email.unc.edu (M.A.S.)

**Keywords:** MerTK, positron emission tomography, fluorine-18, radiolabeling, cancer

## Abstract

MerTK (Mer tyrosine kinase), a receptor tyrosine kinase, is ectopically or aberrantly expressed in numerous human hematologic and solid malignancies. Although a variety of MerTK targeting therapies are being developed to enhance outcomes for patients with various cancers, the sensitivity of tumors to MerTK suppression may not be uniform due to the heterogeneity of solid tumors and different tumor stages. In this report, we develop a series of radiolabeled agents as potential MerTK PET (positron emission tomography) agents. In our initial in vivo evaluation, [^18^F]-**MerTK-6** showed prominent uptake rate (4.79 ± 0.24%ID/g) in B16F10 tumor-bearing mice. The tumor to muscle ratio reached 1.86 and 3.09 at 0.5 and 2 h post-injection, respectively. In summary, [^18^F]-**MerTK-6** is a promising PET agent for MerTK imaging and is worth further evaluation in future studies.

## 1. Introduction

MerTK, a receptor tyrosine kinase of the TAM (TYRO3, AXL, and MERTK) family, is over-expressed or ectopically expressed in a wide variety of cancers [1,2], including acute lymphoblastic leukemia (ALL) [3], non-small cell lung cancer (NSCLC) [4], melanoma [5], prostate cancer [6], glioblastoma [7], etc. In fact, MerTK mediates the activation of several canonical oncogenic signaling pathways in cancer cells [8,9]. In addition, due to the important physiological role of MerTK in the innate immune system, MerTK inhibitors may potentially reduce tumor growth by changing the immunosuppressive environment and stimulating antitumor immunity [10,11]. Indeed, based on the important functions of MerTK, many MerTK targeted therapies are in development to enhance outcomes for patients with a variety of types of cancers, and a few are in clinical trials [12]. Despite the enthusiasm, tumor sensitivity to MerTK suppression may not be uniform due to the heterogeneity of solid tumors and different disease stages (for example, primary v. metastatic disease) [13,14]. Clearly, there is an urgent need to better predict which cancer patients are likely to respond to such novel interventions, as well as monitor the therapeutic responses. Although the drug metabolism study based on Mass analysis could provide information on biodistribution and metabolism of small pharmaceutical molecules in vivo [15], PET is a non-invasive imaging technology that can quantitatively evaluate biological targets or biochemical processes in vivo [16,17,18,19]. Nevertheless, research on MerTK targeted PET agent are very limited [20]. Therefore, the aim of this research is to develop radio-labeled agents that will allow us to directly measure MerTK expression and distribution during different disease stages, non-invasively and repetitively.

We have been committed to the development of novel therapeutics against MerTK for an extended period and have developed several small-molecule MerTK inhibitors with great potency and different selectivity profiles [21,22,23,24,25]. UNC5293 is a new MerTK-specific inhibitor developed recently at UNC, which is extremely potent against MerTK (Ki is 0.19 nM) and very selective against the kinome (Ambit selectivity score S_50_ = 0.041 at 100 nM) [25]. Since target specificity is one of the key requirements of PET agents, the discovery of UNC5293A provides us with a solid foundation for developing MerTK PET ligands.

In this research, we developed a series of potential MerTK PET agents based on the core of UNC5293 (UNC6429/UNC5650) and evaluated their use in B16F10 tumor-bearing mice.

## 2. Results and Discussion

### 2.1. Chemistry

**Scheme 1:**
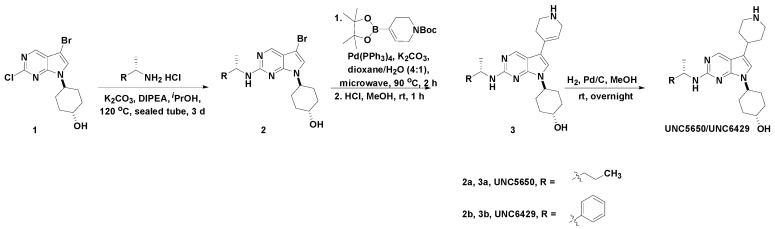
The synthesis of MerTK-target molecule core UNC6429 and UNC 5650.

As shown in Scheme 1**,** UNC6429 and UNC5650 were synthesized using a three-step sequence. Generally, the starting material **1** was heated with an appropriate primary amine (commercially available and enantiomerically pure) in a sealed tube under basic conditions for 3 days to complete the S_N_Ar replacement reaction. After purification, the resulting intermediate **2** underwent a Suzuki coupling reaction, followed by deprotection of the Boc group with hydrogen chloride to afford us with intermediate **3**. Finally, UNC6429 and UNC5650 were prepared by hydrogenation of the double bond using palladium on carbon with overall yields of 33% and 62%, respectively.

**Scheme 2:**
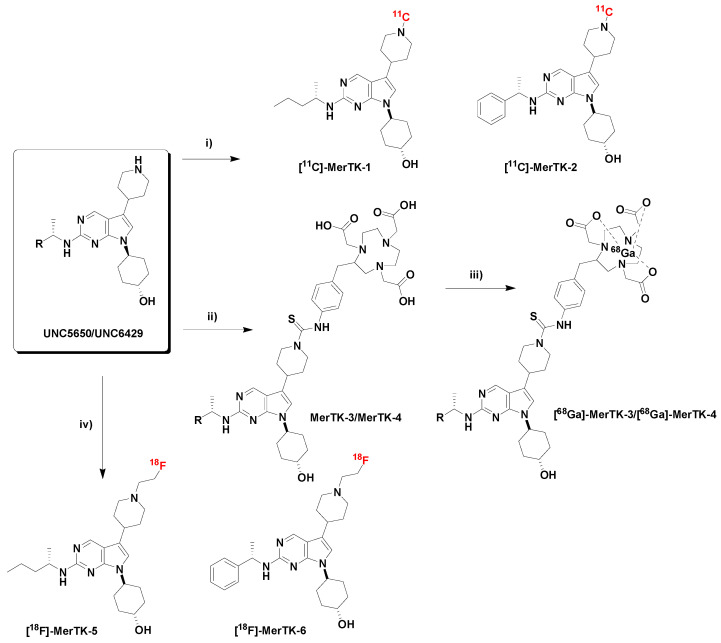
Labeling protocols of MerTK target molecules UNC 5650 and UNC6429 with carbon-11, gallium-68 and fluorine-18. (i) [^11^C]-methyl iodide, DMSO, K_2_CO_3_, heating. 28%RCY; (ii) NOTA-Bn-NCS, acetonitrile. 76% yield; (iii) Sodium acetate buffer (0.5 M), [^68^Ga]-GaCl_3_ in 0.04 M HCl, heating at 80 °C for 10 min. 75–82% RCY; (iv) [^18^F]-2-fluoroethyl 4-methylbenzenesulfonate, K_2_CO_3_, acetonitrile, 110 °C 15 min. 46.0–60.4% RCY.

UNC6429 and UNC5650 were then used to form corresponding precursors and standards according to different labeling protocols, as shown in Scheme 2. Route 1 focuses on C-11 labeling. The standards [^12^C]-**MerTK-1** and [^12^C]-**MerTK-2** were synthesized from UNC6429 and UNC5650, respectively, after methylation with methyl iodide. The same reaction was used to produce C-11 labeled PET agents when [^11^C]-MeI was used as the reagent. Route 2 introduced chelators for radiometal labeling. The precursors **MerTK-3** and **MerTK-4** were prepared by reacting UNC6429 or UNC5650 with NOTA-Bn-NCS. Precursors **MerTK-3** and **MerTK-4** were purified by semi-preparative HPLC and their structures were confirmed by Mass spectrum. Route 3 involved fluorination. The standards of [^19^F]-**MerTK-5** and [^19^F]-**MerTK-6** were prepared by a nucleophilic substitution reaction with [^19^F]-2-fluoroethyl 4-methylbenzenesulfonate.

The inhibitory activities of standards towards MerTK, Axl, Tyro3 and Flt3 were determined in our in-house microcapillary electrophoresis (MCE) assays [25]. As presented in Table 1, the primary targets of these compounds are all MerTK.

### 2.2. Radiochemistry

With the precursors and standards in hand, we explored their radiolabeling with easily available positron nuclides: carbon-11, Gallium-68, and Fluorine-18. C-11 labeled **MerTK-1** and **MerTK-2** were obtained with lower yields due to the difficulty in HPLC purification (the precursor and the product had close retention times). The short half-life of ^11^C (t_1/2_ = 20.4 min) added more challenges: only one HPLC purification could be done for each reaction. The IC_50_ value of **MerTK-1** and **MerTK-2** against MerTK were determined to be 4.2 nM and 61 nM, respectively (Table 1). Good selectivity over Axl, Tyro3 and Flt3 was observed. The ^68^Ga (half-life of 67.6 min and up to 1.89 MeV positron energy) could label **MerTK-3** and **MerTK-4** efficiently; however, the initial pilot study in mice did not provide promising results (<1%ID/g tumor uptakes were observed). Therefore, we did not measure their binding affinity and focused on developing fluorine-18 labeled PET agents for MerTK imaging due to its relatively long half-life (109.8 min) and high resolution (up to 0.64 MeV positron energy) on the PET imaging.

**Figure 1:**
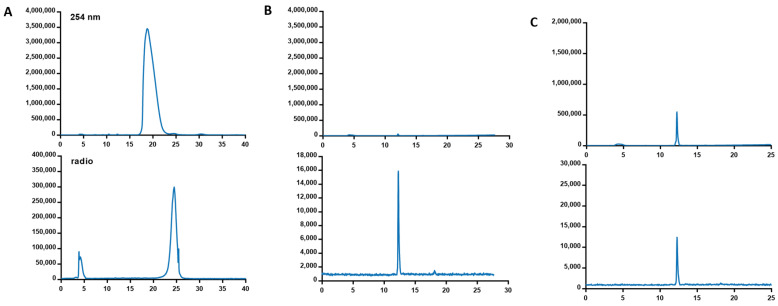
HPLC condition: Column: Phenomenex, Gemini 5 μm C18 110A, New Column 250 × 4.6 mm. Solvent A: 0.1%TFA water; Solvent B: 0.1%TFA acetonitrile; Flow rate: 1 mL/min, column temperature: 19 to 21 °C. (**A**) 0 to 2 min: isocratic 5% solvent B, 2 to 35 min: isocratic 18% solvent B; (**B**) reinjection of [^18^F]-**MerTK-6**: 0 to 2 min: isocratic 5% solvent B, 2 to 22 min: 5–95% solvent B, 22 to 35 min: isocratic 95% solvent B; (**C**) Co-injection of [^18^F]-**MerTK-6** with [^19^F]-**MerTK-6**: 0 to 2 min: isocratic 5% solvent B, 2 to 22 min: 5–95% solvent B, 22 to 35 min: isocratic 95% solvent B.

As shown in Scheme 2, the fluorine-18 labeling on UNC 5650 and UNC6429 were carried out using a two-step sequence. First, [^18^F]-2-fluoroethyl 4-methylbenzenesulfonate was freshly prepared by heating the ethylene ditosylate with anhydrous [^18^F]-tetrabutylammonium fluoride ([^18^F]-TBAF) in anhydrous acetonitrile at 110 °C for 15 min, followed by purification using radio-HPLC. The collected fraction containing [^18^F]-2-fluoroethyl 4-methylbenzenesulfonate was loaded on a Sep-Pak C18 cartridge, washed with 10 mL water, and then eluted with 1 mL anhydrous acetonitrile. The elution was concentrated under evaporation. Then UNC 6429 (2 mg) was added to react with purified [^18^F]-2-fluoroethyl 4-methylbenzenesulfonate under the basic condition at 110 °C for 15 min. The desired products ([^18^F]-**MerTK-5** and [^18^F]-**MerTK-6**) were purified on radio-HPLC and followed by reformulation. The identity of the final product was confirmed by co-injection with the standard compound in HPLC. The IC_50_ value of **MerTK-5** and **MerTK-6** were determined to be 15 nM and 37 nM against MerTK, respectively, with good selectivity over Axl, Tyro3 and Flt3 (Table 1). Although **MerTK-5** had a higher binding affinity towards MerTK, the initial PET study suggested that **MerTK-6** had more prominent tumor uptake and contrast. Therefore, we focused on **MerTK-6** in the initial evaluation. The HPLC spectra in Figure 1 illustrate the purification and quality control of [^18^F]-**MerTK-6**.

### 2.3. Evaluation of the LogP

In order to evaluate the hydrophilicity and lipophilicity of this fluorine-18 labeled agent [^18^F]-**MerTK-6**, we measured the 1-octanol/water partition coefficient (Log*P*) of [^18^F]-**MerTK-6**. The resulting fractions were counted using a gamma counter. The reaction was repeated three times. The log*P* values of [^18^F]-**MerTK-6** (1.56 ± 0.02) showed that it was moderately lipophilic, indicating that it had good cell membrane permeability and tumor cell uptake potential.

### 2.4. PET Imaging Study on Mice

**Figure 2:**
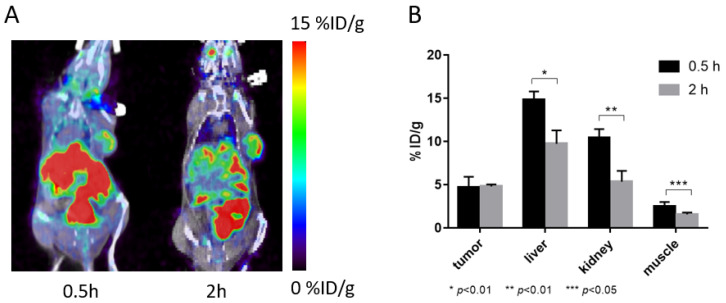
PET images evaluation of [^18^F]-**MerTK-6** in nude mice bearing B16F10 tumors. (**A**) Representative PET image at 0.5 h and 2 h post-injection; (**B**) Major organ and tumor uptakes of [^18^F]-**MerTK-6** at 0.5 h and 2 h post-injection (three mice per group).

Evaluation of the PET agent [^18^F]-**MerTK-6** was performed on B16F10 tumor-bearing mice. Representative images and main organ uptakes are shown in Figure 2. At 30 min post-injection (p.i), the uptake in tumor, liver, kidney, and muscle was 4.65 ± 1.25, 14.79 ± 0.99, 10.42 ± 1.01, and 2.51 ± 0.48%ID/g, respectively. The uptake at 2 h p.i. was 4.79 ± 0.24, 9.72 ± 1.56, 5.34 ± 1.25, and 1.55 ± 0.23%ID/g, respectively. Overall, the tumor uptake was maintained at ~5%ID/g and the tumor to muscle contrast increased to 3.09 at 2 h p.i, compared with 1.86 at 0.5 h p.i. No apparent tumor uptake was observed when [^68^Ga]-**MerTK-3** or [^68^Ga]-**MerTK-4** was injected into the B16F10 tumors.

## 3. Materials and Methods

### 3.1. Chemistry

Microwave reactions were carried out using a CEM Discover-S reactor with a vertically focused IR external temperature sensor and an Explorer 72 autosampler. The dynamic mode was used to set up the desired temperature and hold time with the following fixed parameters: PreStirring, 1 min; Pressure, 200 psi; Power, 200 W; PowerMax, off; Stirring, high. Flash chromatography was carried out on Teledyne ISCO Combi Flash^®^ R_f_ 200 with pre-packed silica gel disposable columns. Preparative HPLC (Agilent Technologies 1260 Infinity, Santa Clara, CA, USA) was performed with UV detection at 220 or 254 nm. Samples were injected onto a 75 × 30 mm, 5 μm, C18(2) column at room temperature. The flow rate was 30 mL/min. Various linear gradients were used with solvent A (0.1% TFA in water) and solvent B (0.1% TFA in acetonitrile). Analytical HPLC was performed with a prominence diode array detector (Shimadzu SPD-M20A, Kyoto, Japan). Samples were injected onto a 3.6 µm PEPTIDE XB-C18 100 Å, 150 × 4.6 mm LC column at room temperature. The flow rate was 1.0 mL/min. Analytical thin-layer chromatography (TLC) was performed with silica gel 60 F_254_, and 0.25 mm pre-coated TLC plates. The TLC plates were visualized using UV_254_ and phosphomolybdic acid with charring. All ^1^H NMR spectra were obtained with a 400 MHz spectrometer (Agilent VnmrJ, Santa Clara, CA, USA) using CDCl_3_ (7.26 ppm), or CD_3_OD (2.05 ppm) as an internal reference. Signals are reported as m (multiplet), s (singlet), d (doublet), t (triplet), q (quartet), p (pentet), and bs (broad singlet); and coupling constants are reported in hertz (Hz). The ^13^C NMR spectra were obtained with a 100 MHz spectrometer (Agilent VnmrJ, Santa Clara, CA, USA) using CDCl_3_ (77.2 ppm), or CD_3_OD (49.0 ppm) as the internal standard. Representative NMR spectrums were provided in Supplementary Material. LC/MS (Agilent Technologies 1260 Infinity II, Santa Clara, CA, USA) was performed using an analytical instrument with the UV detector set to 220 nm, 254 nm, and 280 nm, and a single quadrupole mass spectrometer using an electrospray ionization (ESI) source. Samples were injected (2 μL) onto a 4.6 × 50 mm, 1.8 μm, C18 column at room temperature. A linear gradient from 10% to 100% B (0.1% acetic acid in MeOH) in 5.0 min was followed by pumping 100% B for another 2 or 4 min with A being H_2_O + 0.1% acetic acid. The flow rate was 1.0 mL/min. The purity of all final compounds (>95%) was determined by LC-MS.

#### 3.1.1. Synthesis of UNC5650

General procedure A [25].

A mixture of **1** (3.30 g, 10.0 mmol); (*S*)-pentan-2-amine (3.48 g, 40.0 mmol); potassium carbonate (5.52 g, 40.0 mmol); and *N*,*N*-diisopropylethylamine (7.0 mL, 40.0 mmol) in *i*PrOH (80 mL) was heated at 120 °C for 3 d. The reaction mixture was extracted between EtOAc (3 × 80 mL) and H_2_O (80 mL). The combined organic layers were washed with brine (50 mL), dried (Na_2_SO_4_), filtered, and concentrated under reduced pressure. The residue was purified by an ISCO silica gel column to afford the desired product **2** as a pale-yellow solid (2.82 g, 74%). ^1^H NMR (400 MHz, CD_3_OD) δ 8.26 (s, 1H); 7.12 (s, 1H), 4.45 (ddd, *J* = 11.5, 10.3, 4.5 Hz, 1H); 4.07 (dd, *J* = 13.1, 6.6 Hz, 1H); 3.65 (ddd, *J* = 11.0, 7.7, 4.3 Hz, 1H), 2.12–1.83 (m, 6H); 1.68–1.37 (m, 6H), 1.21 (dd, *J* = 6.9, 2.7 Hz, 3H); 0.94 (dd, *J* = 9.0, 5.4 Hz, 3H); ^13^C NMR (100 MHz, CD_3_OD) δ 160.32; 153.26,;150.13; 123.29; 112.24; 88.86; 70.22; 54.27; 47.71; 40.21; 35.31; 35.30; 31.46; 31.32; 21.05; 20.54; 14.45. MS (ESI) for [M + H]^+^ (C_17_H_26_BrN_4_O^+^): calcd. *m/z* 381.12; found *m/z* 381.11.; LC-MS: 98% purity.

A suspension of **2** (381 mg, 1.0 mmol), Pd(PPh_3_)_4_ (58 mg, 0.05 mmol), 3,6-dihydro-2*H*-pyridine-1-*N*-Boc-4-boronic acid, pinacol ester (618 mg, 2.0 mmol), and potassium carbonate (415 mg, 3.0 mmol) in a mixture of dioxane and H_2_O (4:1, 10 mL) was heated at 90 °C under microwave radiation for 2.0 h. The reaction mixture was cooled to rt and the solvent was removed under reduced pressure. The residue was purified by an ISCO silica gel column to afford a Boc-protected product which was dissolved in MeOH (2.0 mL) and treated with a 4.0 M HCl solution in dioxane (2.0 mL). The resulting solution was stirred at rt for 1 h, and then concentrated under reduced pressure to provide the desired compound **3a** as a pale-yellow solid (276 mg, 72%). ^1^H NMR (400 MHz, CD_3_OD) *δ* 8.76 (s, 1H); 7.66 (s, 1H), 6.17 (s, 1H); 4.56–4.46 (m, 1H); 4.20–4.11 (m, 1H); 3.90–3.85 (m, 2H); 3.72–3.61 (m, 1H); 3.46 (d, *J* = 8.0 Hz, 2H); 2.82–2.75 (m, 2H); 2.15–2.05 (m, 2H); 2.04–1.93 (m, 4H); 1.71–1.58 (m, 2H); 1.53–1.40 (m, 4H); 1.30 (d, *J* = 8.0 Hz, 3H); 0.97 (t, *J* = 8.0 Hz, 3H); ^13^C NMR (100 MHz, CD_3_OD) δ 154.49; 150.48; 138.87; 127.71; 127.10; 116.40; 115.77; 109.18; 72.13; 71.00; 68.50; 60.74; 53.73; 42.38; 41.75; 40.51; 38.09; 33.67; 29.40; 23.13; 19.00; 12.87. MS (ESI) for [M + H]^+^ (C_22_H_34_N_5_O^+^): calcd. *m/z* 384.28; found *m/z* 384.30; LC-MS: 95% purity.

A suspension of **3a** (383 mg, 1.0 mmol) and palladium on carbon (10% Pd, 380 mg) in MeOH (20 mL) was stirred at rt under hydrogen atmosphere overnight. The resulting mixture was filtered through a pad of Celite and the solvent was removed under reduced pressure. The residue was purified by an ISCO silica gel column to afford the desired product **4** (UNC5650) as a yellow solid (240 mg, 62%). ^1^H NMR (400 MHz, CD_3_OD) δ 8.75 (s, 1H), 7.40 (s, 1H), 4.55–4.43 (m, 1H), 4.25–4.10 (m, 1H), 3.75–3.65 (m, 1H), 3.50 (d, *J* = 12.7 Hz, 2H), 3.25–3.10 (m, 3H), 2.22 (d, *J* = 13.9 Hz, 2H), 2.11 (d, *J* = 11.2 Hz, 2H), 2.04–1.86 (m, 6H), 1.73–1.38 (m, 6H), 1.30 (d, *J* = 6.6 Hz, 3H), 0.98 (t, *J* = 7.3 Hz, 3H); ^13^C NMR (100 MHz, CD_3_OD) δ 153.91, 150.43, 137.76, 125.40, 120.05, 110.54, 72.13, 71.00, 68.54, 60.74, 53.38, 43.85, 42.42, 38.12, 33.70, 30.83, 29.51, 29.36, 28.82, 19.10, 18.94, 12.88. MS (ESI) for [M + H]^+^ (C_22_H_36_N_5_O^+^): calcd. *m/z* 386.29; found *m/z* 386.30; LC-MS: 95% purity.

#### 3.1.2. Synthesis of UNC6429

The title compound UNC6429 was synthesized according to the general procedure A as a yellow solid (240 mg, 0.523 mmol). ^1^H NMR (400 MHz; CD_3_OD) δ 8.70 (s, 1H); 7.47–7.41 (m, 2H); 7.33 (dd, *J* = 15.9, 8.0 Hz, 3H); 7.28–7.20 (m, 1H); 5.10 (q, *J* = 7.0 Hz, 1H); 4.37–4.25 (m, 1H); 3.66 (tt, *J* = 10.9; 4.2 Hz, 1H); 3.49 (d, *J* = 13.5 Hz, 2H); 3.21–3.04 (m, 3H); 2.19 (d, *J* = 14.3 Hz, 2H); 2.12–1.98 (m, 2H); 1.86 (tdt, *J* = 16.0, 12.6, 8.2 Hz, 5H); 1.72–1.58 (m, 4H); 1.45 (tt, *J* = 12.9, 10.6 Hz, 2H); MS (ESI) for [M + H^+^] (C_25_H_34_N_5_O^+^): calcd. *m/z* 420.28; found *m/z* 420.30; LC-MS 99% purity.

#### 3.1.3. Synthesis of **MerTK-1**

General procedure B.

The synthesis of **MerTK-1** was modified with literature method [25]. To a solution of UNC5650 (19 mg, 49 µmol) and formaldehyde (11 µL, 0.15 mmol, 37%) in dichloromethane (6.0 mL) was added sodium triacetoxyborohydride (85 mg, 0.15 mmol) at rt. After 1 h, the solvent was removed under reduced pressure. The residue was purified by HPLC to afford a Boc protected product which was dissolved in MeOH (1.0 mL) and treated with a 4.0 M HCl solution in dioxane (1.0 mL). After 1 h, the reaction solution was concentrated under reduced pressure to provide **MerTK-1** as a pale-yellow solid (8.0 mg, 41%). ^1^H NMR (400 MHz, CD_3_OD) δ 8.71 (s, 1H); 7.38 (s, 1H); 4.55–4.43 (m, 1H); 4.23–4.10 (m, 1H); 3.75–3.55 (m, 4H); 3.25–3.05 (m, 2H); 2.92 (s, 3H); 2.25 (d, *J* = 14.7 Hz, 2H); 2.15–1.94 (m, 8H); 1.72–1.40 (m, 6H); 1.30 (d, *J* = 6.6 Hz, 3H); 0.98 (t, *J* = 7.2 Hz, 3H); MS (ESI) for [M + H]^+^ (C_23_H_38_N_5_O^+^): calcd. *m/z* 400.31; found *m/z* 400.35; LC-MS: 95% purity.

#### 3.1.4. Synthesis of **MerTK-2**

**MerTK-2** was synthesized according to the general procedure B as a yellow solid (17 mg, 39 µmol) in 60% yield. ^1^H NMR (400 MHz, CD_3_OD) δ 8.72 (s, 1H); 7.44 (d, *J* = 7.8 Hz, 2H); 7.36–7.32 (m, 3H); 7.24 (t, *J* = 7.8 Hz, 1H); 5.11 (q, *J* = 6.7 Hz, 1H); 4.35–4.28 (m, 1H); 3.69–3.58 (m, 3H); 3.17 (t, *J* = 11.5 Hz, 2H); 3.10–3.04 (m, 1H); 2.91 (s, 3H); 2.25–2.20 (m, 2H); 2.08–1.78 (m, 7H); 1.68–1.63 (m, 1H); 1.64 (d, *J* = 8.1 Hz, 3H); 1.52–1.38 (m, 2H); MS (ESI) for [M + H]^+^ (C_26_H_36_N_5_O^+^): calcd. *m/z* 434.29; found *m/z* 434.30; LC-MS: 95% purity.

#### 3.1.5. Synthesis of **MerTK-3**/**MerTK-4**

**MerTK-3** was synthesized by modifying the literature method [26]. Generally, the UNC5650 (1.0 equiv.) was reacted with NOTA-Bn-NCS (2.0 equilv.) under the basic condition to yield **MerTK-3** after purification by HPLC. The collected product was lyophilized and Mass spectrum confirmed. MS (ESI), calcd. for C_42_H_62_N_9_O_7_S (M + 1H), 836.45; found, 836.70.

The same method was applied to synthesize precursor **MerTK-4** (5.1 mg, 56%) with UNC6429 as the starting material. ^1^H NMR (500 MHz, CD_3_OD) δ 8.67 (s, 1H); 7.48–7.42 (m, 2H), 7.40–7.19 (m, 8H); 5.11 (q, *J* = 6.9 Hz, 1H); 4.91 (overlapping with CD_3_OD peak, 1H); 4.36–4.27 (m, 1H); 4.07–3.84 (m, 3H); 3.78–3.53 (m, 3H); 3.50–3.04 (m, 11H); 2.98–2.59 (m, 6H); 2.08–2.02 (m, 3H); 1.92–1.66 (m, 6H); 1.65 (d, *J* = 7.0 Hz, 3H); 1.54–1.40 (m, 2H); 1.36–1.27 (m, 2H); MS (ESI), calcd for C_45_H_61_N_9_O_7_S (M + 2H), 871.44; found, 871.43).

#### 3.1.6. Synthesis of **MerTK-5**

General procedure C.

The synthesis of MerTK was modified from literature method [25]. To a solution of UNC5650 (10.0 mg, 21.8 μmol) and 2-fluoroethyl 4-toluenesulfonate (3.7 μL, 22 μmol) in acetonitrile (2.2 mL) was added sodium iodide (1.6 mg, 11 μmol), and sodium carbonate (10.4 mg, 98.2 μmol). The reaction mixture was heated at 65 °C for 18 h and concentrated in vacuo. The residue was purified by normal phase chromatography (dichloromethane/methanol gradient) to afford the desired compound **MerTK-5** as a pale-yellow oil, which was freeze dried to give an orange solid (4.0 mg, 9.3 μmol) in 43% yield. ^1^H NMR (400 MHz, CD_3_OD) δ 8.53 (s, 1H); 7.01 (s, 1H); 4.94–4.91 (m, 1H); 4.83–4.77 (m, 1H); 4.50–4.39 (m, 1H); 4.15–4.05 (m, 1H); 3.73–3.63 (m, 4H); 3.59–3.47 (m, 2H); 3.15–3.05 (m, 1H); 2.26 (d, *J* = 14.3 Hz, 2H); 2.14–1.94 (m, 8H); 1.70–1.57 (m, 1H); 1.57–1.40 (m, 6H); 1.24 (d, *J* = 6.5 Hz, 3H); 0.97 (t, *J* = 7.2 Hz, 3H); MS (ESI) for [M + H^+^] (C_24_H_39_FN_5_O^+^): calcd. *m/z* 432.31; found *m/z* 432.30; LC-MS 96% purity.

#### 3.1.7. Synthesis of Compound **MerTK-6**

**MerTK-6** was synthesized according to the general procedure C as an orange foam (5.6 mg, 12 μmol) in 49% yield. ^1^H NMR (400 MHz, CD_3_OD) δ 8.48 (s, 1H); 7.43–7.36 (m, 2H); 7.27 (dd, *J* = 8.4, 6.9 Hz, 2H); 7.20–7.11 (m, 1H); 6.83 (s, 1H); 5.02 (q, *J* = 7.0 Hz, 1H); 4.78 (t, *J* = 4.7 Hz, 1H); 4.67 (t, *J* = 4.7 Hz, 1H); 4.29–4.19 (m, 1H); 3.69–3.60 (m, 1H); 3,46–3.32 (m, 2H); 3.20–3.05 (m, 2H); 2.93–2.82 (m, 1H); 2.80–2.63 (m, 2H;, 2.21–1.75 (m, 8H); 1.72–1.65 (m, 1H); 1.54 (d, *J* = 7.0 Hz, 3H); 1.51–1.36 (m, 2H); 1.35–1.25 (m, 1H); MS (ESI) for [M + H^+^] (C_27_H_37_FN_5_O^+^): calcd. *m/z* 466.30; found *m/z* 466.30; LC-MS 98% purity.

#### 3.1.8. Microcapillary Electrophoresis (MCE) Assays 

Microcapillary Electrophoresis (MCE) Assays were performed with literature method [25]. These assays were performed in a 384-well polypropylene microplate in a final volume of 50 µL of 50 mM Hepes at Ph 7.4 containing 10 mM MgCl_2_, 1.0 mM DTT, 0.01% Triton X-100, 0.1% Bovine Serum Albumin (BSA), 1.0 µM fluorescent substrate (Table 2), and ATP at the Km for each enzyme (Table 2). All reactions were terminated by addition of 20 µL of 70 mM EDTA. After an 180 min incubation, phosphorylated and unphosphorylated substrate peptides (Table 2) were separated in buffer supplemented with 1 x CR-8 on a LabChip EZ Reader equipped with a 12-sipper chip. Data were analyzed using EZ Reader software.

### 3.2. Radiochemistry

General procedure D.

Radiolabeling protocol for [^18^F]-**MerTK-6**: [^18^F]-2-fluoroethyl 4-methylbenzenesulfonate was prepared (50–60 mCi) as previously described by us [27]. Then K_2_CO_3_ (2 mg), UNC6429 (2 mg) and 60 μL acetonitrile were added in and heated at 90 °C for 20 min. The resulting tracer was purified by HPLC to get [^18^F]-**MerTK-6** with a radiochemistry yield (RCY) of 46.0% based on the last step. The collected solution containing [^18^F]-**MerTK-6** was diluted with 5 mL milliQ pure water and loaded onto a Sep-Pak C18 cartridge. The C18 cartridge was washed with 10 mL of water, and then the product was washed out by 1 mL acetonitrile. After removing the acetonitrile under vacuum, the product was diluted with saline. The radiochemical purity (>99%) of the final product was checked on HPLC with the condition as: Phenomenex, Gemini 5 μm C18 110A, New Column 250 × 4.6 mm. Solvent A: 0.1%TFA water; Solvent B: 0.1% TFA acetonitrile; Flow rate: 1 mL/min, column temperature: 19 to 21 °C. Zero to two min: isocratic 5% solvent B, 2 to 22 min: 5–95% solvent B, 22 to 35 min: isocratic 95% solvent B. The structure of the product was confirmed by co-injection of [^18^F]-**MerTK-6** with [^19^F]-**MerTK-6** ([^19^F]-UNC7333). The [^18^F]-**MerTK-5** was also synthesized according to general procedure D by using UNC5650 as a starting material.

### 3.3. Evaluation of LogP

The Log*P* value of the [^18^F]-**MerTK-6** was calculated by the gamma particle counts of samples in the aqueous phase or 1-octanol phase by Automatic Gamma Counter 2480-0010 (PerkinElmer Instruments Inc., Waltham, MA, USA).

The [^18^F]-**MerTK-6** was collected after HPLC purification. After reformulation (pH value around 7.4), 20 µL [^18^F]-**MerTK-6** sample in saline was added to the mixture of 1 mL Mili-Q® water and 1 mL 1-octanol in a 5 mL Eppendorf tube. The tube was shacked thoroughly and then let stand still for 5 min. Then the 100 µL 1-octanol phase and 100 µL aqueous phase were subjected to a gamma counter separately and the gamma counts were recorded (*n* = 3). The Log*P* value was then calculated and expressed as a mean value± standard derivation.

### 3.4. Mouse Model

All animal studies were reviewed and approved by The University of North Carolina at Chapel Hill Institutional Animal Care and Use Committee. The B16F10 tumor cell was obtained from the LCCC tissue culture facility (the University of North Carolina at Chapel Hill, Chapel Hill, NC, USA). The B16F10 tumor-bearing nude mouse model was prepared as described previously [28]. Briefly, B16/F10 cells were subcutaneously injected on the right flank of C57BL/6 female mice (Jackson Laboratory). The tumor volume was measured daily. When the tumor size reached 100 mm^3^, the mice were used for PET imaging studies.

### 3.5. PET Imaging

B16F10 tumor-bearing mice (*n* = 3/group) were intravenously injected via the tail vein with tracers. At 30 min and 120 min post-injection, a 10-min static emission scan was acquired with a SuperArgus small-animal PET/CT scanner. The regions of interests (ROIs) were drawn over the tumor and other organs and calculated as %ID/g. The mean uptake and standard deviation were calculated.

## 4. Conclusions

In this study, we synthesized several MerTK targeted PET agents based on the core structure of MerTK-specific inhibitor UNC5293. Of them, [^18^F]-**MerTK-6** showed a significant uptake rate (4.79 ± 0.24%ID/g) in B16F10 tumor-bearing mice. At 0.5 and 2 h after injection, the tumor to muscle ratio reached 1.86 and 3.09, respectively. In summary, [^18^F]-**MerTK-6** is a promising PET agent for MerTK imaging and worthy of further evaluation in future studies. There are a few MerTK inhibitors entered into clinical trials recently, such as MRX-2843 [3], INCB081776 [29], and RXDX-106 [30]. The MerTK-target PET imaging tracer would potentially help evaluating target engagement and adjusting treatment plan for individual patient.

## Figures and Tables

**Table 1:** TAM and Flt3 inhibitory activities of standards.

Compound	Structure	MerTK IC_50_ (nM) *^a^*	Axl IC_50_ (nM) *^a^*	Tyro3 IC_50_ (nM) *^a^*	Flt3 IC_50_ (nM) *^a^*
**MerTK-1**	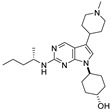	4.2	370	65	850
**MerTK-2**	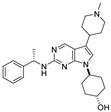	61	1700	180	>30,000
**MerTK-4**	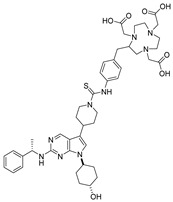	13	4100	180	>30,000
**MerTK-5**	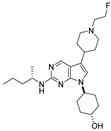	15	1100	190	1000
**MerTK-6**	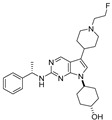	37	2100	120	5500

*^a^* Values are the mean of two or more independent assays.

**Table 2:** Assay conditions for MCE assays.

Kinase	Peptide Substrate	Kinase (nM)	ATP (µM)
Mer	5-FAM-EFPIYDFLPAKKK-CONH_2_	1.7	22.3
Axl	5-FAM-KKKKEEIYFFF-CONH_2_	16	200
Tyro3	5-FAM-EFPIYDFLPAKKK-CONH_2_	5	40
Flt3	5-FAM-KKKKEEIYFFF-CONH_2_	0.3	275

## Data Availability

Not applicable.

## Supplementary Materials

The following are available online. Figures S1–S7: ^1^H NMR spectra for standard compounds.

## Sample Availability

Not available.
