# Supplementary material for: The Synthesis and Initial Evaluation of MerTK Targeted PET Agents
Source: Molecules. 2022 Feb 22;27(5):1460. doi: 10.3390/molecules27051460 (PMC8911752; doi:10.3390/molecules27051460)
Supplement: Supplementary file 1 [file molecules-27-01460-s001.zip › molecules-1543170-supplementary.pdf]

Supporting Information

The Synthesis and Initial Evaluation of MerTK Targeted PET Agents

Li Wang[1,†], Yubai Zhou[2,†], Xuedan Wu[1], Xinrui Ma[1], Bing Li[2], Ransheng Ding[2], Michael A. Stashko[2], Zhanhong Wu[1,]⋆, Xiaodong Wang[2,]⋆, and Zibo Li[1]

Contents

Figure.S1: ^{1}H NMR spectra of **UNC5650** 2

Figure.S2: ^{1}H NMR spectra of **UNC6429** 3

Figure.S3: ^{1}H NMR spectra of **MerTK-1** 4

Figure.S4: ^{1}H NMR spectra of **MerTK-2** 5

Figure.S5: ^{1}H NMR spectra of **MerTK-4** 6

Figure.S6: ^{1}H NMR spectra of **MerTK-5** 7

Figure.S7: ^{1}H NMR spectra of **MerTK-6** 8

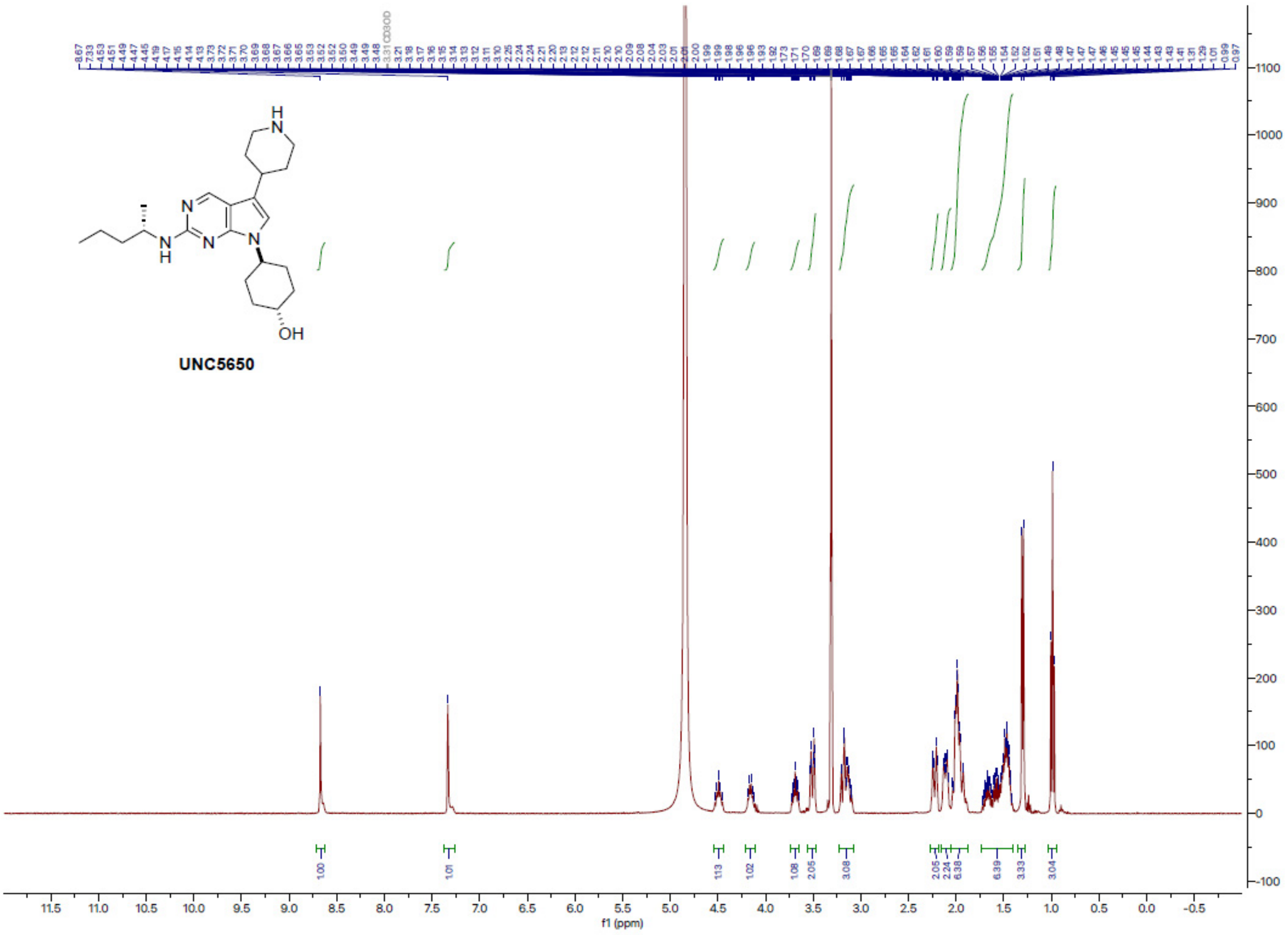

Figure S1 ^{1}H NMR spectra of **UNC5650**

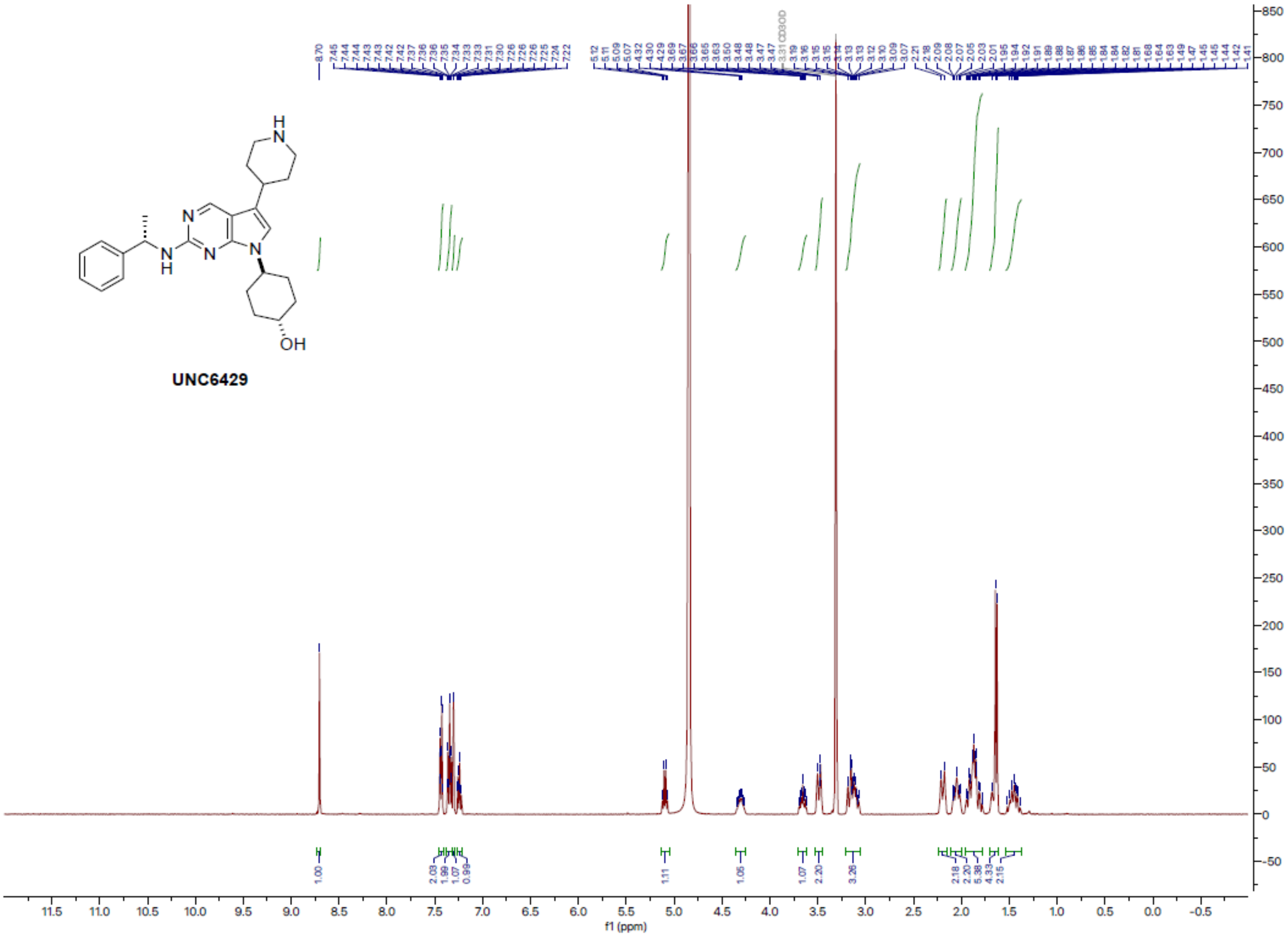

Figure S2 ^{1}H NMR spectra of **UNC6429**

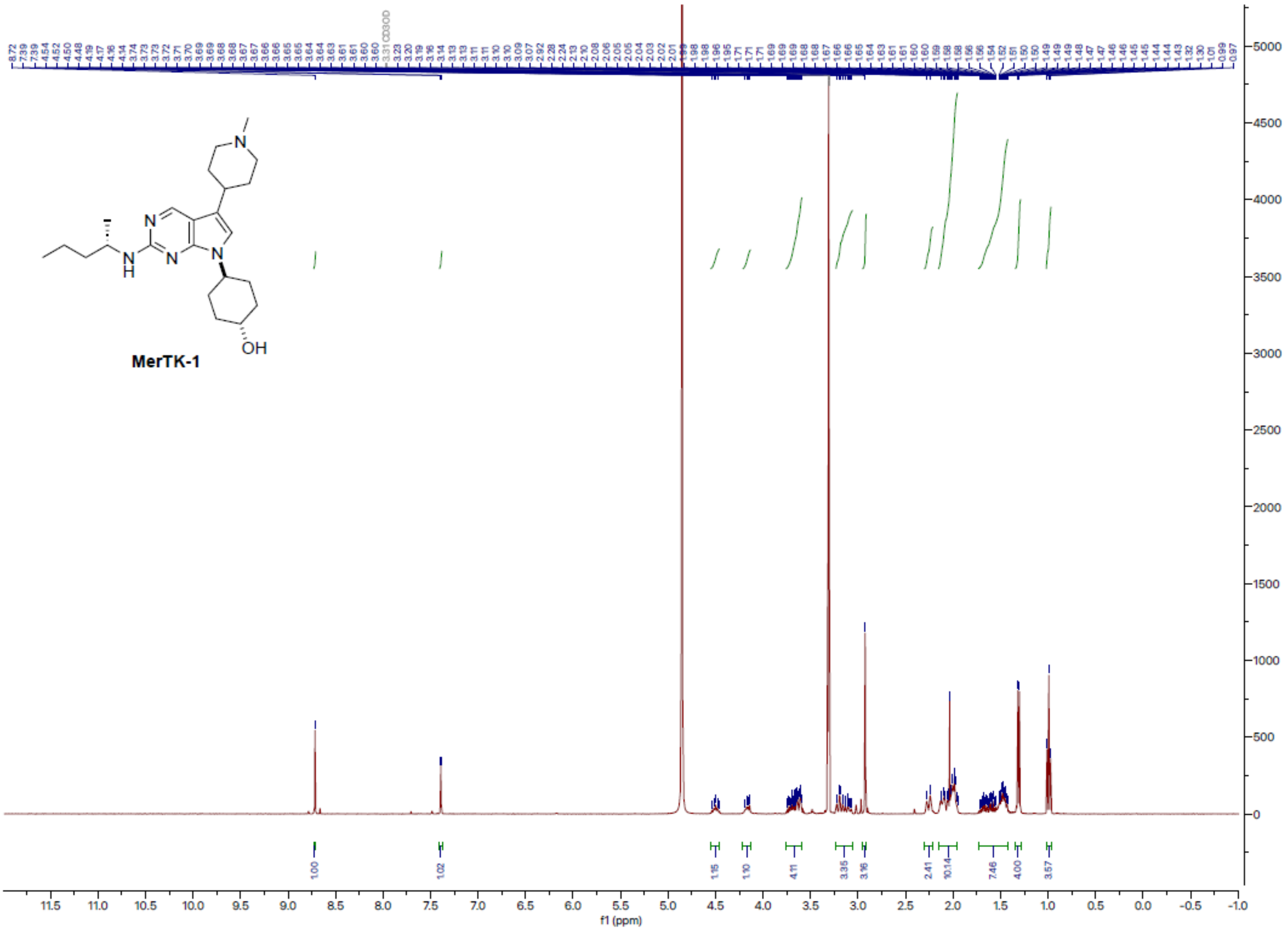

Figure S3 [1] H NMR spectra of **MerTK-1**

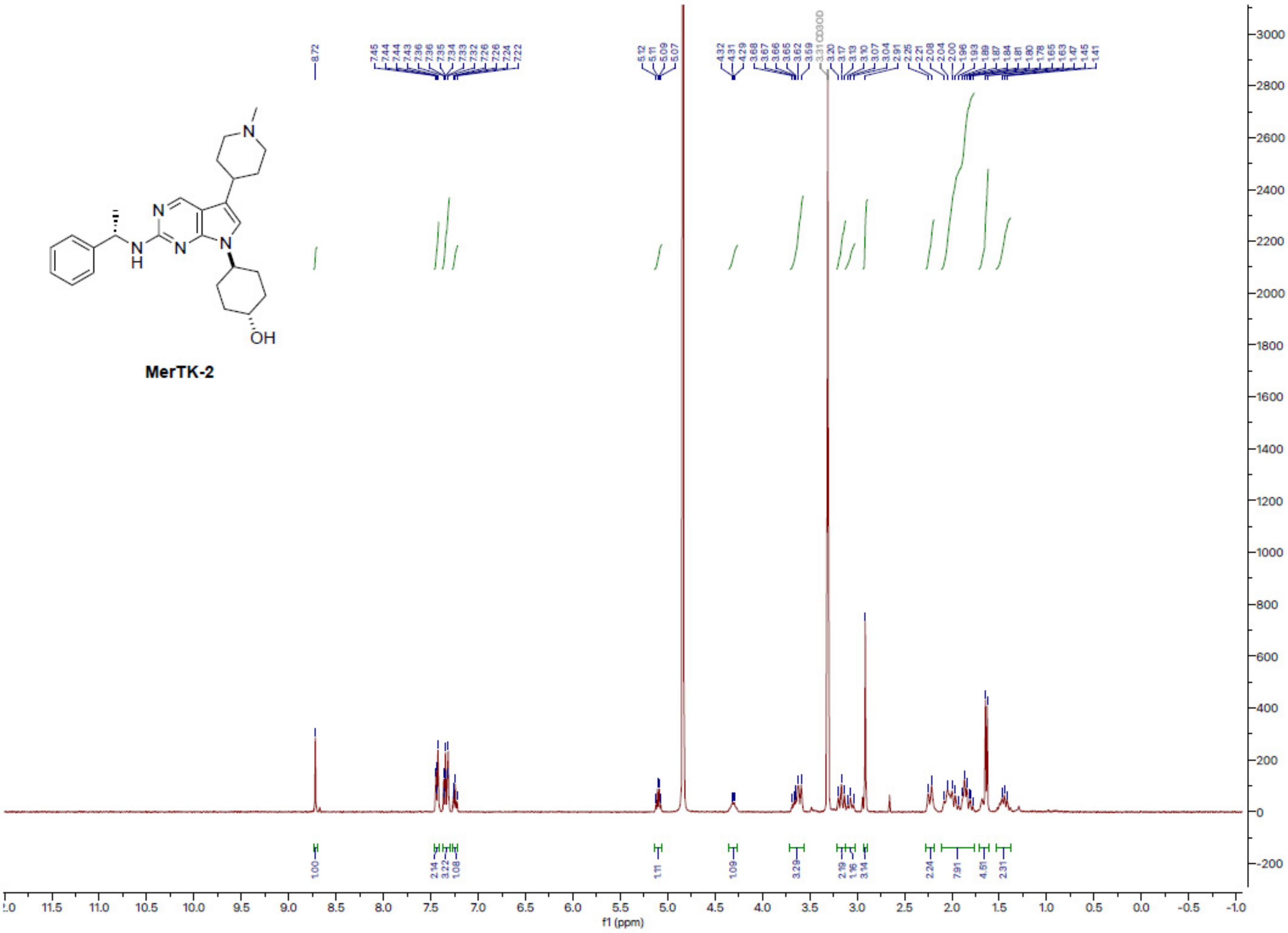

Figure S4 [1] H NMR spectra of **MerTK-2**

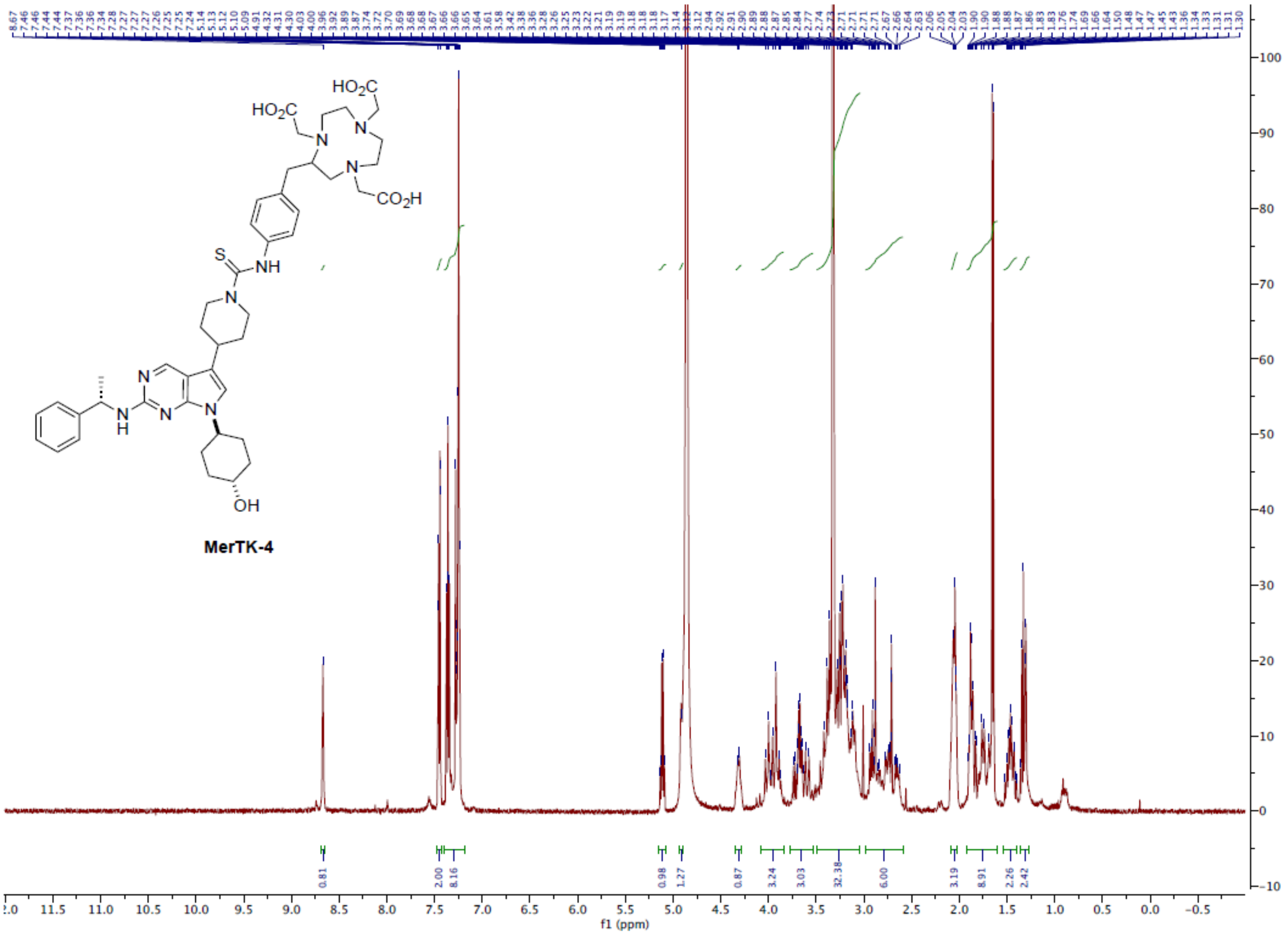

Figure S5 [1] H NMR spectra of **MerTK-4**

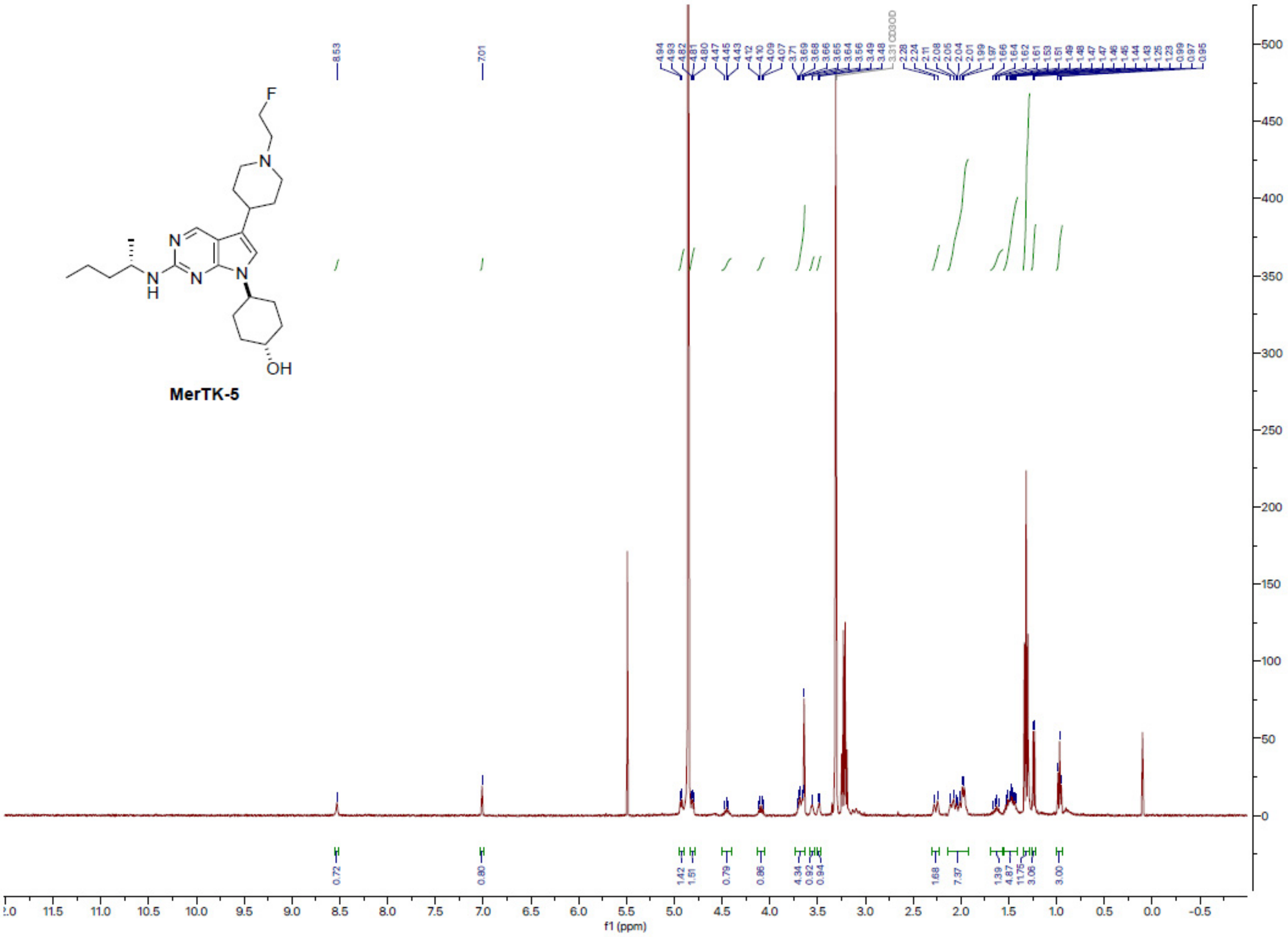

Figure S6 [1] H NMR spectra of **MerTK-5**

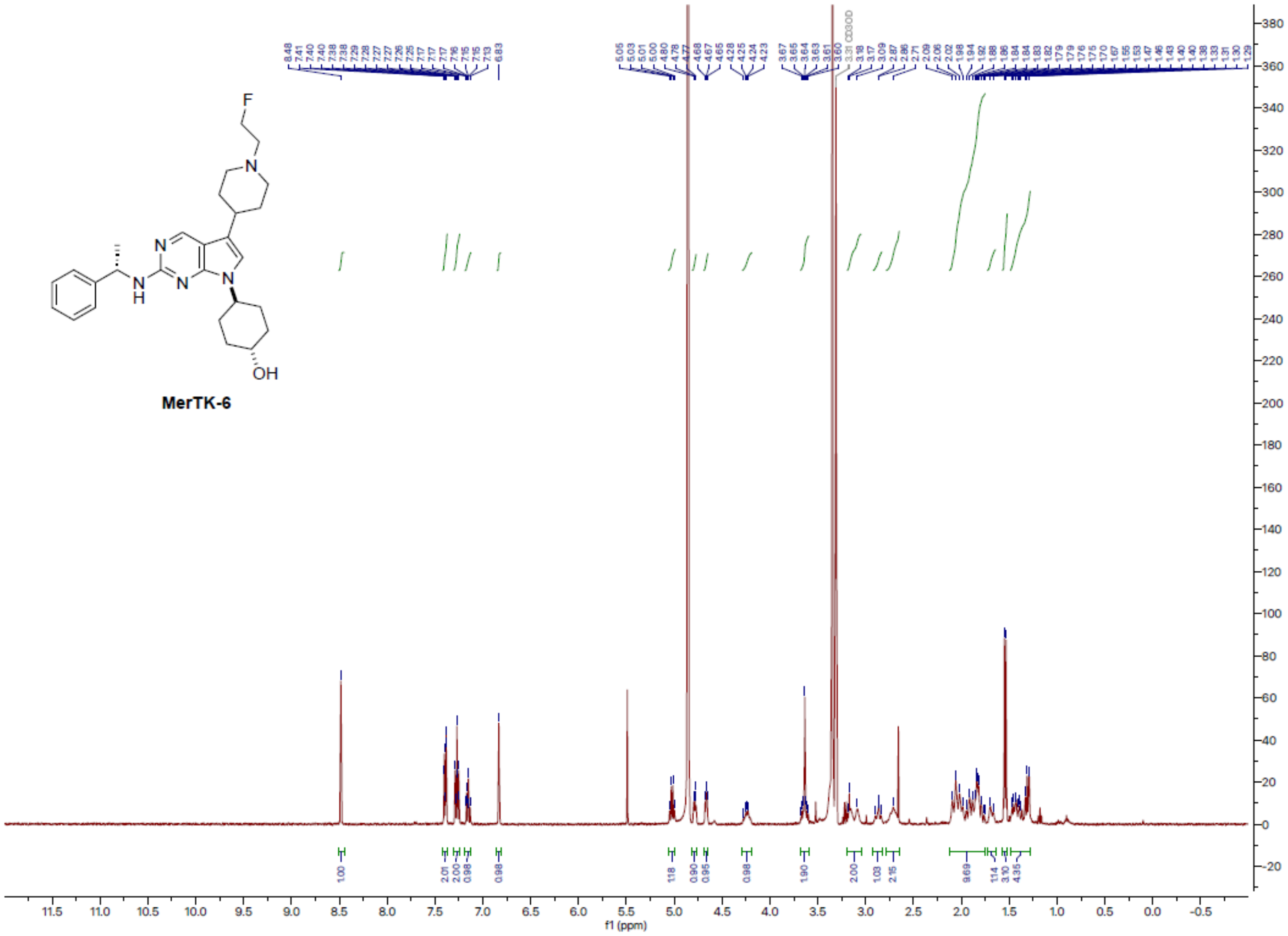

Figure S7 ^{1}H NMR spectra of **MerTK-6**
